# Identification of the *agr* Peptide of *Listeria monocytogenes*

**DOI:** 10.3389/fmicb.2016.00989

**Published:** 2016-06-22

**Authors:** Marion Zetzmann, Andrés Sánchez-Kopper, Mark S. Waidmann, Bastian Blombach, Christian U. Riedel

**Affiliations:** ^1^Institute of Microbiology and Biotechnology, University of UlmUlm, Germany; ^2^Institute of Biochemical Engineering, University of StuttgartStuttgart, Germany; ^3^CEQIATEC, Costa Rica Institute of TechnologyCartago, Costa Rica

**Keywords:** autoinducing peptide, accessory gene regulator, *Listeria monocytogenes*, peptide sensing

## Abstract

*Listeria monocytogenes* (*Lm*) is an important food-borne human pathogen that is able to strive under a wide range of environmental conditions. Its accessory gene regulator (*agr*) system was shown to impact on biofilm formation and virulence and has been proposed as one of the regulatory mechanisms involved in adaptation to these changing environments. The *Lm agr* operon is homologous to the *Staphylococcus aureus* system, which includes an *agrD*-encoded autoinducing peptide that stimulates expression of the *agr* genes via the AgrCA two-component system and is required for regulation of target genes. The aim of the present study was to identify the native autoinducing peptide (AIP) of *Lm* using a luciferase reporter system in wildtype and *agrD* deficient strains, rational design of synthetic peptides and mass spectrometry. Upon deletion of *agrD*, luciferase reporter activity driven by the P_II_ promoter of the *agr* operon was completely abolished and this defect was restored by co-cultivation of the *agrD*-negative reporter strain with a producer strain. Based on the sequence and structures of known AIPs of other organisms, a set of potential *Lm* AIPs was designed and tested for P_II_-activation. This led to the identification of a cyclic pentapeptide that was able to induce P_II_-driven luciferase reporter activity and restore defective invasion of the *agrD* deletion mutant into Caco-2 cells. Analysis of supernatants of a recombinant *Escherichia coli* strain expressing AgrBD identified a peptide identical in mass and charge to the cyclic pentapeptide. The *Lm agr* system is specific for this pentapeptide since the AIP of *Lactobacillus plantarum*, which also is a pentapeptide yet with different amino acid sequence, did not induce P_II_ activity. In summary, the presented results provide further evidence for the hypothesis that the *agrD* gene of *Lm* encodes a secreted AIP responsible for autoregulation of the *agr* system of *Lm*. Additionally, the structure of the native *Lm* AIP was identified.

## Introduction

The Gram-positive bacterium *Listeria monocytogenes* (*Lm*) is an opportunistic, intracellular pathogen that may cause severe, food-borne infections in high-risk groups such as immunocom promised persons, elderly people and pregnant women (Freitag et al., 2009). *Lm* is able to survive and replicate in a wide range of environments including soil, various food products, and different niches inside its human host (Freitag et al., 2009; Vivant et al., 2013; Ferreira et al., 2014; Gahan and Hill, 2014). In order to adapt to these changing conditions, *L. monocytogenes* possesses 15 complete two-component systems (Williams et al., 2005) and a number of regulatory circuits (Guariglia-Oropeza et al., 2014). The accessory gene regulator (*agr*) locus encodes one of these systems and has been shown to be involved in biofilm formation, virulence and survival in the environment (Autret et al., 2003; Rieu et al., 2007; Riedel et al., 2009; Vivant et al., 2015).

The prototype *agr* system was described for *S. aureus* and consists of the four gene operon *agrBDCA* (Novick and Geisinger, 2008). Of the four proteins encoded by the *agr* operon, AgrB is a membrane-bound peptidase that cleaves and processes the *agrD*-derived propeptide at the C-terminus, catalyzes formation of a thiolactone ring with a central cysteine, and, in combination with the signal peptidase SpsB, effects export and release of the active autoinducing peptide (AIP). Upon accumulation in the extracellular space, this AIP activates a two-component system consisting of AgrC (receptor-histidine kinase) and AgrA (response regulator). Expression of the operon is driven by the P_II_ promoter upstream of *agrB* and is subject to autoregulation via AgrA. Target genes of the staphylococcal *agr* system are either directly regulated by AgrA or by a regulatory RNAIII transcribed in the opposite direction from the P_III_ promoter adjacent to P_II_ (Thoendel et al., 2011).

Homologous *agr* systems have been identified in a number of Gram-positive microorganisms including streptococci, clostridia, lactobacilli, *Bacillus sp.*, and *Enterococcus faecalis* (Wuster and Babu, 2008). The effects of *agr* regulation are pleiotropic. In *S. aureus*, the *agr* system regulates a wide range of genes involved in biofilm formation, virulence, and immune evasion (Queck et al., 2008; Thoendel et al., 2011). The *agr* system of *Lactobacillus plantarum* is involved in regulation of cell morphology and adhesion to glass surfaces (Sturme et al., 2005; Fujii et al., 2008). Similar to the staphylococcal system, the *agr*-like *fsr* system of *E. faecalis* and the *agr* system of *Lm* are involved in regulation of biofilm formation and virulence (Autret et al., 2003; Rieu et al., 2007; Riedel et al., 2009; Cook and Federle, 2014). Moreover, in *Lm* more than 650 genes are directly or indirectly regulated by the *agr* system as shown by transcriptional profiling of an *agrD* deletion mutant (Riedel et al., 2009). This suggests that *agr* systems represent rather global regulatory mechanisms.

Despite similarities on protein level, genetic organization, and phenotypic traits regulated, known *agr* systems differ regarding their mechanisms of target gene regulation. While in staphylococci, a significant number of *agr*-dependent genes are regulated by RNAIII (Thoendel et al., 2011), no information on RNAIII transcripts are available in other organisms. In *E. faecalis* and *Lm*, the genetic information upstream of the *agr* operon differs from that of staphylococci in that the preceding gene is transcribed in the same direction as the *agr* genes and no putative P_III_ promoters have been identified (Qin et al., 2001; Autret et al., 2003). Moreover, despite extensive bioinformatic approaches or transcriptional profiling a regulatory RNAIII has not been identified in *Lm* (Mandin et al., 2007; Toledo-Arana et al., 2009; Mellin and Cossart, 2012; Wurtzel et al., 2012). This suggests that in *Lm* (and *E. faecalis*) target genes are regulated by AgrA and/or other transcriptional regulators affected by AgrA-dependent regulation. However, it can not be excluded that the AIP signals through other two-component system besides AgrCA.

Structural information of AIPs is available only for a limited number of species. In *S. aureus*, four *agr* specificity groups with different AIPs varying in size from 7 to 9 amino acids (aa) are known (Novick and Geisinger, 2008). Similarly, three *agr* specificity groups exist in *S. epidermidis* with AIPs of 8–12 aa (Otto et al., 1998; Olson et al., 2014). The AIP of *S. intermedius* and *S. lugdunensis* are 9 and 7 aa in size, respectively (Ji et al., 1997; Kalkum et al., 2003). Outside the genus *Staphylococcus*, AIPs have been characterized for *E. faecalis* (11 aa), *L. plantarum* (5 aa), and *C. acetobutylicum* (6 aa) (Nakayama et al., 2001; Sturme et al., 2005; Steiner et al., 2012). Most of the known AIPs contain a thiolactone ring formed by the 5 C-terminal aa. Exceptions are the AIPs of *C. acetobutylicum* and *E. faecalis*, which have ring structures consisting of 6 and 9 aa, respectively (Nakayama et al., 2001; Steiner et al., 2012). Another common feature is a central cysteine, which is replaced by a serine in some cases, required for thiolactone ring formation.

For staphylococci, *E. faecalis* and *Lm*, a contribution of the *agr* system to virulence gene regulation has been demonstrated and *agr*-deficient mutants are attenuated (Riedel et al., 2009; Thoendel et al., 2011; Cook and Federle, 2014). Consequently, interference with *agr* signaling was proposed as a therapeutic approach (Gray et al., 2013). Of note, the specificity of the interaction between the AIP and its cognate receptor AgrA has been used to device improved strategies by fusing the AIP to a bacteriocin to induce lysis of the targeted bacteria (Qiu et al., 2003). The structure of the native AIP of *Lm* has not been elucidated so far. With the present study, we aim closing this gap in order to further elucidate the components and mechanisms of the *agr* autoregulatory circuit of *Lm* and to facilitate future studies on strategies to interfere with cell–cell communication of this important human pathogen.

## Materials and Methods

### Bacterial Strains and Culture Conditions

All strains and plasmids used in this study are listed in **Table 1**. *L. monocytogenes* was generally incubated in Brain Heart Infusion broth (BHI, Oxoid Ltd) at 30°C. *E. coli* strains were grown in lysogeny broth (LB). For solid media, 15 g/l agar were added to the broth before autoclaving. Antibiotics were added if necessary. Where appropriate, kanamycin was used at a final concentration of 50 (for *E. coli* strains) and 15 μg/ml chloramphenicol were used for both species. For *Lm* strains carrying a chromosomal copy of pPL2 derivatives chloramphenicol was used at 7 μg/ml.

**Table 1:** Bacterial strains and plasmids used in the present study.

Strain/plasmid	Characteristics	Reference/source
**Strains**
*Escherichia coli* DH10B	Cloning host	Thermo Fisher Scientific
*E. coli* BL21 DE3	Used for protein overexpression	New England Biolabs
*E. coli* BL21 DE3 pET29a_*agrB*	IPTG-inducible expression of *agrB*, Kan^r^	This study
*E. coli* BL21 DE3 pET29a_*agrBD*	IPTG-inducible expression of *agrBD*, Kan^r^	This study
*Listeria monocytogenes* EGD-e		Bécavin et al., 2014
*L. monocytogenes* Δ*agrD*	In-frame deletion of *agrD* in strain EGD-e	Riedel et al., 2009
*L. monocytogenes*Δ*agrD*::pIMK2*agrD*	pIMK2*agrD* integrated into the tRNA^Arg^ locus in the EGD-e chromosome, Kan^r^	Riedel et al., 2009
*L. monocytogenes* EGD-e::pPL2*lux*P_II_	pPL2*luxABCDE*P_II_ integrated into the tRNA^Arg^ locus in the EGD-e chromosome, Cm^r^	This study
*L. monocytogenes* EGD-e *ΔagrD*::pPL2*lux*P_II_	pPL2*lux*P_II_ integrated into the tRNA^Arg^ locus in the EGD-e*ΔagrD* chromosome, Cm^r^	This study
*L. monocytogenes* EGD-e *ΔagrD* pNZ44*agrBD*	Strain with constitutive, P_44_-driven expression of *agrB* and *agrD*, Cm^r^	This study
**Plasmids**
pPL2*lux*	Site-specific integrative vector to study promotor activity in *L. monocytogenes*, Cm^r^	Bron et al., 2006
pPL2*lux*P_II_	Site-specific integrative vector for P_II_ promoter activity analysis, Cm^r^	This study
pNZ44	Plasmid for constitutive gene expression driven from the lactococcal promoter P_44_	McGrath et al., 2001
pNZ44*agrBD*	Plasmid for constitutive P_44_-driven expression of *agrB* and *agrD* in *L. monocytogenes*, Cm^r^	This study
pET29a(+)	Plasmid for strong IPTG inducible expression in *E. coli*, Kan^r^	Merck Millipore
pET29a_*agrB*	IPTG-inducible expression of *agrB* in *E. coli*, Kan^r^	This study
pET29a_*agrBD*	IPTG-inducible expression of *agrBD* in *E. coli*, Kan^r^	This study

### Generation of Recombinant Strains

Primers used for cloning or sequencing purposes are listed in **Table 2**. To study transcriptional activity of the *agr* operon, the P_II_ promoter upstream of *agrB* (Rieu et al., 2007) was amplified with Phusion^®^ polymerase (Thermo Fisher Scientific) using primers PII_fwd_SalI and PII_rev and chromosomal DNA of *Lm* EGD-e wildtype (WT) as template. The obtained PCR fragment was digested with *Sal*I and cloned in frame in front of the luciferase reporter into *Sal*I/*Swa*I-cut pPL2*lux* (Bron et al., 2006). The ligation mix was transformed into *E. coli* ElectroMax^TM^ DH10B (Thermo Fisher Scientific), and the resulting plasmid pPL2*lux*P_II_ was verified by restriction analysis and amplification of the cloned P_II_ promoter using primers PII_fwd_SalI and luxA_rev with subsequent Sanger sequencing of the PCR fragment by a commercial service provider (Eurofins, Germany). The plasmid was transformed into electrocompetent *Lm* EGD-e WT or Δ*agrD* (Riedel et al., 2009) as described previously (Monk et al., 2008) creating *Lm* EGD-e::pPL2*lux*P_II_ and Δ*agrD*::pPL2*lux*P_II_. In both strains, successful chromosomal integration of pPL2*lux*P_II_ at the correct site (tRNA^Arg^) was verified using primers PL95 and PL102 (Lauer et al., 2002).

**Table 2:** Primers used in this study.

Name	Sequence	Reference/source
PII_fwd_SalI	CTGATGTCGACCTTCAAACAGAACAAGACG	This study
PII_rev	CAACTAATTCACCTCCACTAATATTTTACAACG	This study
luxA_rev	TACCTCTGTTTGAGAAAATTGGGGAGG	This study
PL95	ACATAATCAGTCCAAAGTAGATGC	Lauer, 2002
PL102	TATCAGACCTAACCCAAACCTTCC	Lauer, 2002
NZagrBD-fwd	AATTCCATGGGTAATTTTACTGCAAAAGTCCC	This study
NZagrBD-rev	GCATCGAGCTCTTATTTATTTTCGTTTTTTTC	This study
NZ-conf_fwd	CCATACAGGAGAAGGGACGATAGCAA	This study
NZ_colony_rev	CCTTGAGCCAGTTGGGATAGAGC	This study
agrBD_NdeI_fwd	GGAATTCCATATGAGTAATTTTACTGCAAAAGTCCC	This study
agrBD_BamHI_rev	CGCGGATCCATTAATCCTCCACTGTCTAAAATATCTAT	This study

For homologous overexpression of *agrBD*, a PCR fragment containing both genes was amplified using primers NZagrBD_fwd and NZagrBD_rev and chromosomal DNA of *Lm* EGD-e as template. The PCR product was digested with *Nco*I and *Sac*II and ligated as exact transcriptional fusion to the constitutive P_44_ promoter into *Nco*I/*Sac*II digested pNZ44 (McGrath et al., 2001) to yield pNZ44*agrBD*. The product was transformed into *E. coli* DH10B. Clones were screened for plasmid containing the correct insert by PCR using primers NZ-confirm_fwd and NZ_colony_rev and sequencing of the PCR product. The correct plasmid as well as the empty vector (pNZ44) were transformed in electrocompetent *Lm* Δ*agrD* generated as described previously (Monk et al., 2008).

For heterologous AIP production, *agrBD* or *agrB* alone were amplified using primer pairs agrBD_NdeI_fwd/agrBD_BamHI_rev and chromosomal DNA of *Lm* EGD-e WT or Δ*agrD*. Following restriction with *Nde*I and *BamH*I both PCR products were ligated into *Nde*I/*BamH*I digested pET29a(+) (Merck Millipore). This fuses the PCR products to the T7 promoter creating pET29a_*agrB* and pET29a_*agrBD*, respectively. Both plasmids were verified for correct cloning by restriction analysis and Sanger sequencing of inserts.

### Luciferase Reporter Assays

For luciferase reporter assays, growth experiments were performed in white 96-well microtiter plates with transparent bottom (BRANDplates^®^ pureGrade^TM^ S). A single colony was inoculated into BHI and grown over night (o/N; i.e., approx. 16 h). Following o/N growth, cultures were diluted to an optical density at 600 nm (OD_600_) of 0.01 in fresh, sterile BHI. For co-cultivation of AIP producer and reporter strains, o/N cultures of both strains were used to inoculate BHI medium to a OD_600_ of 0.01 and then mixed at a 1:1 ratio. 200 μl aliquots of this mix were transferred into individual wells of the microtiter plates (each condition in triplicates). Plates were incubated at 30°C in a Tecan Infinite M200 plate reader and OD_600_ and luminescence intensity were measured every hour.

### Synthetic Peptides

Synthetic peptides were purchased from Peptide Protein Research Ltd (UK) in lyophilized form with >70% purity. Peptides were reconstituted in dimethyl sulfoxide (DMSO) at 2 mM and stored at -20°C until further use. For experiments, these stocks were diluted as appropriate in 25% (v/v) DMSO in phosphate-buffered saline (PBS) to give the final concentrations as indicated. To test the effect of peptides on P_II_ activity, reporter strains (*Lm* EGD-e::pPL2*lux*P_II_ or Δ*agrD*::pPL2*lux*P_II_) were grown o/N and diluted to an OD_600_ of 0.01 in fresh BHI. 180 μl aliquots were distributed in 96 well microtiter plates (each condition in triplicate) and incubated at 30°C for 2 h. At this stage, 20 μl of diluted peptides were added to obtain the indicated final concentrations (5 nM–50 μM) and plates were incubated at 30°C in a Tecan Infinite M200 plate reader with hourly OD_600_ and luminescence intensity measurements.

### AIP Production in *E. coli*

For heterologous AIP production, pET29a_*agrB* or pET29a_*agrBD* were transformed into *E. coli* BL21(DE3) (New England Biolabs) and transformants were selected on LB agar containing kanamycin. Four single colonies were streaked onto two LB agar plates containing kanamycin with or without 1 mM isopropyl β-D-1-thiogalactopyranoside (IPTG). A clone showing good growth in the absence of IPTG but reduced growth in its presence was selected and a single colony was inoculated into 5 ml of LB medium and grown o/N on a rotary shaker at 37°C. Using the o/N culture, 500 ml LeMaster and Richards minimal medium (Paliy and Gunasekera, 2007) containing 50 mM glucose were inoculated to a final OD_600_ of 0.1 and incubated on a rotary shaker at 37°C to an OD_600_ of 0.8. At this stage, expression was induced by addition of 1 mM ITPG. Following incubation under the same conditions for an additional 2 h, bacterial cells were pelleted via centrifugation (3000 × g, 30 min and 4°C) and supernatants were collected, filter sterilized, frozen in liquid nitrogen and lyophilized. Lyophilized samples were stored at -20°C until further analysis by LC–MS/MS.

### LC–MS/MS Analysis

The lyophilized supernatants of recombinant *E. coli* strains were reconstituted in a 25:35:35:5 H_2_O:Isopropanol:CH_3_CN:HCOOH mixture and diluted 1:10 in H_2_O. 5 μl were injected into a reverse-phase column with corresponding guard column (Aeris^TM^ PEPTIDE 3.6u XB-C18 150 × 2.1 mm, Security Guard^TM^ ULTRA 2 × 2.1 mm guard column, Phenomenex). A constant flow rate of 0.4 ml/min was applied. Mobile phase A consisted of water with 0.2% (v/v) formic acid and mobile phase B was acetonitrile with 0.2% (v/v) formic acid. Elution program was: isocratic hold at 5% B for 5 min followed by a linear gradient from 5 to 45% B over 80 min. After each sample, the column was washed with 90% B for 10 min and equilibrated at starting conditions. Data was obtained in positive auto MS/MS mode on an Agilent 6540 Accurate-Mass Quadrupole (LC-Q-TOF/MS) with ESI Jet Stream Technology using the following conditions: drying gas flow rate of 10 l/min with a gas temperature of 250°C, nebulizer with 40 lb per square inch gauge, sheath gas flow rate of 10 l/min, sheath gas temperature of 300°C, capillary voltage of 4000 V, and fragmentor voltage of 170 V. The collision energy was set by formula with 4.5 slope and 10 offset. Data analysis was performed using Mass Hunter Workstation Software (Ver.B.05.519.0, Agilent Technologies) and the “Find compounds by formula” algorithms. Synthetic peptides were analyzed using the same conditions as the recombinant peptides expressed in *E. coli* to compare retention time, accurate mass and fragmentation patterns.

### Invasion Assay

Invasion of *Lm* into Caco-2 cells was tested using a standard gentamycin protection assay essentially as described previously (Riedel et al., 2009). Briefly, Caco-2 cells were cultured in DMEM supplemented with 10% (v/v) fetal calf serum (FCS), 10 mM L-glutamine, 1% (v/v) penicillin/streptomycin and 1% (v/v) non-essential amino acids (NEAA) at 37°C and a 5% CO_2_ atmosphere. Cells were seeded to a density of 2 × 10^5^ cells per well in a 24 well plate and cultivated to a monolayer for 4 days. One day prior to the experiment, culture media without antibiotics was added. A fresh o/N culture of the indicated bacterial strains was diluted 1:10 in 10 ml fresh BHI and grown to mid-exponential phase (OD_600_ = 0.8). Where appropriate, peptide R5T0 was added (5 μM final concentration). Bacteria were pelleted and diluted in DMEM containing 10 mM L-glutamine and 1% NEAA to 10^8^ colony forming units per ml (cfu/ml) (OD_600_ = 0.5). 1 ml of this suspension was added to Caco-2 cells in quadruplicates (MOI = 100). Cells were incubated for 1 h to allow invasion of bacteria. To kill remaining extracellular bacteria, cells were washed once with PBS and 1 ml DMEM containing 10 μg/ml gentamicin (Gibco^®^) was added to the cells. After 1 h of incubation, cells were washed twice with PBS, lysed with ice-cold water and cfu/ml were determined by plating serial dilutions on BHI agar.

### Statistical Analysis

All experiments were conducted in at least three biological replicates. Results were analyzed by Student’s *t*-test or ANOVA with Bonferroni post-test analysis to correct for multiple comparisons using GraphPad Prism (version 6) as indicated in figure legends and Supplementary Data Sheet 1. Differences between different strains or conditions were considered statistically significant at *p* < 0.05.

## Results

### P_II_-Activity in *Lm* EGD-e

**FIGURE 1:**
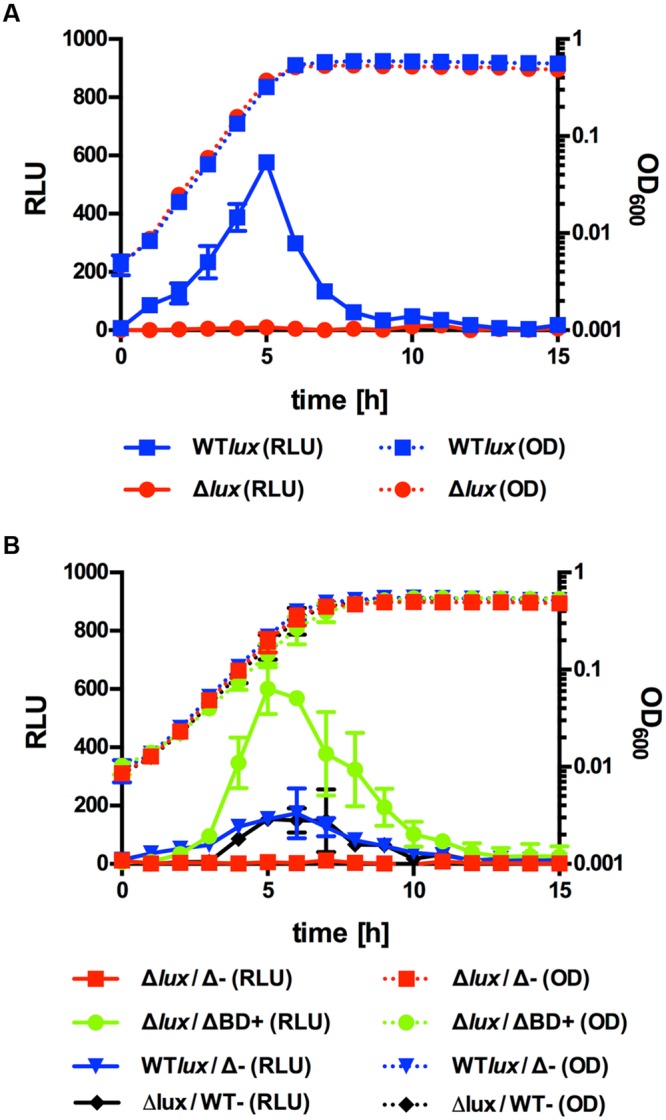
**Growth (OD_600_) and luminescence (relative luminescence units; RLU) of *Lm* EGD-e::pPL2*lux*P_II_ (WT*lux*) or Δ*agrD*::pPL2*lux*P_II_ (Δ*lux*) grown alone **(A)** or in co-culture **(B)** with Δ*agrD* pNZ44*agrBD* (ΔBD+), Δ*agrD* pNZ44 (Δ-), or EGD-e pNZ44 (WT-).** Values are mean ± standard deviation of three independent experiments. Statistical analysis of RLU values was performed by Student’s *t*-test and results are provided in Supplementary Data Sheet 1.

P_II_ promoter activity was analyzed in *Lm* EGD-e::pPL2*lux*P_II_ and Δ*agrD*::pPL2*lux*P_II_ during growth in BHI medium at 30°C (**Figure 1A**). No differences in growth or final OD_600_ were observed between the two strains ruling out an effect of growth on luciferase activity. In the WT background, a significant increase in P_II_-dependent luciferase activity was observed during exponential growth with a peak in late exponential phase. By contrast, no luminescence above background could be detected for the *agrD*-deficient strain throughout the experiment. This suggests that the AIP is required for transcriptional activity of P_II_.

AIPs are usually secreted into the extracellular environment. In order to confirm that the AIP of *Lm* is acting as an extracellular peptide, similar growth experiments were conducted using co-incubation of AIP producer and reporter strains in different combinations (**Figure 1B**). As expected, the *agrD*-deficient reporter strain showed no P_II_ activity when incubated with *Lm* EGD-e Δ*agrD*. However, high levels of luminescence were observed using the same reporter strain in combination with *Lm* Δ*agrD* pNZ44*agrBD*, a Δ*agrD* derivative expressing *agrBD* from the P44 promoter on pNZ44. Luminescence in this setup was significantly higher compared to co-cultures of the WT reporter with the *agrD* deletion mutant or the *agrD*-deficient reporter strain with *Lm* EGD-e pNZ44 (i.e., the empty vector control) suggesting that AIP levels produced by *Lm* Δ*agrD* pNZ44*agrBD* are higher than that of the WT.

### P_II_ Activation by Synthetic AIP Candidates

**FIGURE 2:**
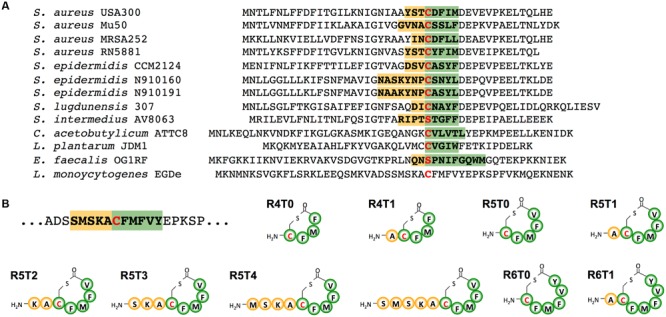
**(A)** Amino acid sequences of AgrD propetides with known structure compared with the AgrD sequence of *Listeria monocytogenes*. **(B)** Structure of synthetic cyclic peptides tested for autoinducing activity in *L. monocytogenes*. Amino acid residues of the native **(A)** or synthetic **(B)** peptides involved in thiolactone ring formation are labeled in green, those found in the N-terminal tails in yellow. The central cysteine or serine is marked by a red letter.

**FIGURE 3:**
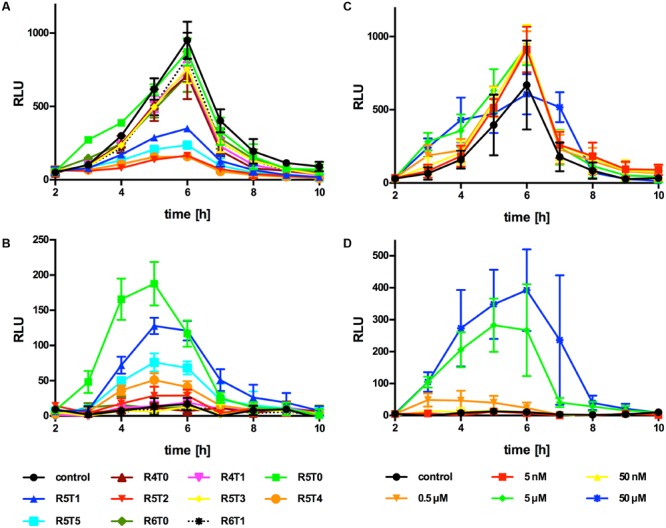
**Luminescence (RLU) of *Lm* EGD-e::pPL2*lux*P_II_**(A,C)** or Δ*agrD*::pPL2*lux*P_II_**(B,D)** grown in the presence of different synthetic peptides (**A,B**; peptide concentration: 5 μM) or peptide R5T0 at the indicated concentrations **(C,D)**.** Values are mean ± standard deviation of three independent experiments. Statistical analysis of RLU values was performed by ANOVA and Bonferroni test to correct for multiple comparisons and results are provided in Supplementary Data Sheet 1.

Upon several attempts we were unable to identify the active AIP in supernatants of *Lm* EGD-e WT or the AIP overproducing strain Δ*agrD* pNZ44*agrBD* grown in either BHI or modified Welshimer’s broth. Sequence alignment of AIPs with a resolved structure, revealed that most AIPs consist of a 5 aa thiolactone ring with N-terminal tail varying from 0 to 7 aa (**Figure 2A**). Using this information, a range of peptides based on the AgrD sequence of *Lm* EGD-e were synthesized consisting of a thiolactone ring of 4–6 aa and an N-terminal tail of 0–5 aa (**Figure 2B**). The effect of these peptides on P_II_-driven luciferase activity was tested using the reporter strains *Lm* EGD-e::pPL2*lux*P_II_ and Δ*agrD*::pPL2*lux*P_II_. At 5 μM, none of the peptides had a measurable effect on growth of the reporter strains (Supplementary Figures S1A,B). The peptide R5T0 consisting of a 5 aa thiolactone ring with no N-terminal tail slightly increased P_II_-driven luminescence in the WT reporter strain during the first 4 h of the experiment (**Figure 3A**). However, at later stages luminescence was comparable to the control, i.e., reporter without peptide. Interestingly, some of the tested peptides (R5T1, R5T2, R5T4, and R5T5) significantly inhibited luminescence of the WT reporter strain. More importantly, some of the peptides (R5T0, R5T1, R5T4, and R5T5) induced luminescence by the Δ*agrD* reporter strain (**Figure 3B**). The most potent inducer of P_II_ activity was the peptide R5T0, i.e., a cyclic pentapeptide with the amino acid sequence Cys-Phe-Met-Phe-Val (CFMFV). At concentration of 5 and 50 μM, R5T0 also induced luminescence above control levels during the first 4 h in the WT reporter (**Figure 3C**) and for up to 7 h in the *agrD*-deficient reporter (**Figure 3D**). This suggests that the most likely candidate for the native AIP of *Lm* EGD-e is the peptide R5T0.

### The Synthetic AIP Restores the Invasion Defect of *Lm ΔagrD*

**FIGURE 4:**
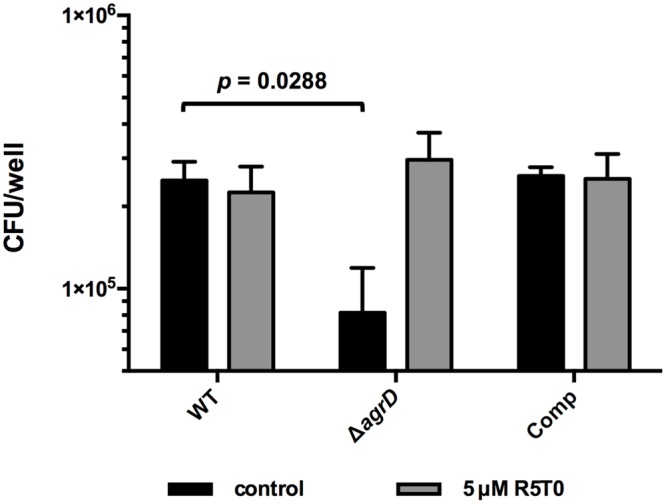
**Invasion of *Lm* EGD-e (WT), EGD-e Δ*agrD* (Δ*agrD*), or EGD-e Δ*agrD*::pIMK2*agrD* (Comp) into Caco-2 cells.** Bacteria were grown either in the absence (black bars) or presence of 5 μM peptide R5T0. Values are colony forming units per well (cfu/well) and are mean ± standard deviation of three independent experiments. Statistical analysis was performed by comparing all strains for a given condition (with or without peptide) by ANOVA. Bonferroni post tests were used to adjust *p* values for multiple comparisons.

Deletion of Δ*agrD* and thus lack of a functional AIP results in reduced promoter activity of virulence factors and attenuated virulence (Riedel et al., 2009). In order to check if R5T0 is not only able to induce P_II_ activity but also functionally complement the Δ*agrD* mutant, invasion assays were performed with *Lm* EGD-e Δ*agrD* grown in the presence and absence of R5T0 (**Figure 4**). As observed previously, deletion of *agrD* results in reduced invasion into Caco-2 intestinal epithelial cells and this defect was genetically complemented by integration of pIMK2*agrD*, i.e., a plasmid for constitutive expression of *agrD* (Riedel et al., 2009). More importantly, growth in the presence of 5 μM R5T0 completely restored invasion of *Lm* EGD-e Δ*agrD* to WT levels.

### Heterologous Production of the *Lm* AIP in *E. coli*

**FIGURE 5:**
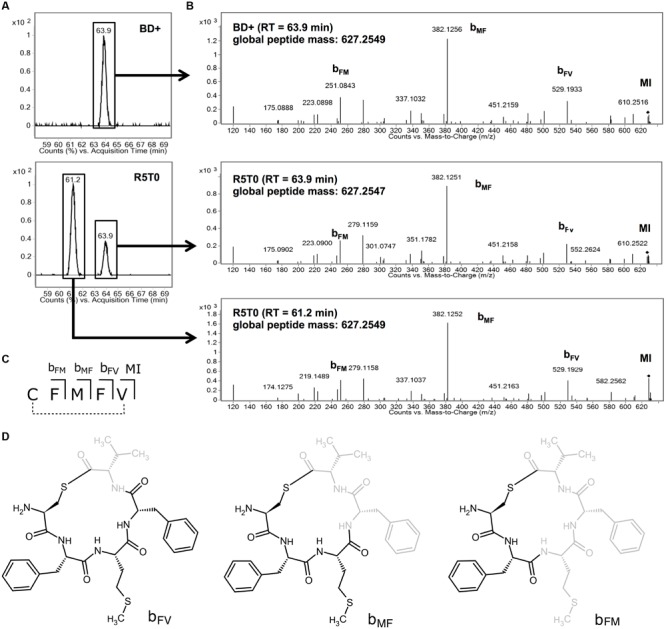
**(A)** Extracted ion chromatograms performed on culture supernatant of *Escherichia coli* BL21 DE3 pET29a_*agrBD* after induction with IPTG (BD+; upper panel) and the synthetic peptide R5T0 (lower panel; R5T0 molecular formula: C_31_H_41_N_5_O_5_S_2_). **(B)** Mass spectrometry fragmentation spectra for chromatographic peaks with retention times of 61.2 and 63.9 min (marked with a box in **A**). **(C,D)** Structure and assignment of fragments detected in MS/MS spectra to fragments of R5T0.

In a further approach to identify the AIP of *Lm*, the *agrBD* genes were expressed in *E. coli* using the IPTG-inducible pET29a system. Using LC–MS, a prominent signal was identified in supernatants of an induced culture of *E. coli* BL21 pET29a_*agrBD* (**Figure 5A**) with a mass of 627.2549 (**Figure 5B**). This signal was absent in the non-induced culture or supernatant of a control strain only expressing *agrB* (Supplementary Figure S2). In order to confirm the identity of the overexpressed peptide, analysis of the P_II_-activating synthetic peptide R5T0 was performed. Interestingly, the chromatogram of R5T0 yielded two peaks in close vicinity (**Figure 5A**). Both peaks correspond to peptides with identical mass and fragmentation pattern (**Figure 5B**). However, the different retention times and peak areas indicate that the two peaks represent stereoisomers or conformational isomers at different concentrations.

The peptide present in the supernatant of *E. coli* BL21 pET29a_*agrBD* and both peaks of R5T0 had almost identical global masses (**Figure 5B**). Moreover, all three peptides showed highly similar fragmentation patterns (**Figure 5B**) and several signals of the MS/MS spectra correspond to fragments of R5T0 at a mass accuracy better than 2 ppm (**Table 3**; for corresponding structures see **Figures 5C,D**). These results clearly indicate that the listerial AIP is a cyclic pentapeptide with the amino acid sequence CFMFV forming a thiolactone ring, i.e., the structure of the synthetic peptide R5T0.

**Table 3:** Mass-charge-ratios (*m/z*) of peptide fragments detected by MS/MS and difference to the *m/z* calculated according to the formula of the corresponding R5T0 fragment.

Fragment	Formula	*m/z* (calculated)	*m/z* (measured)^a^	Difference (ppm)	Difference (mDa)
*b*_FV_	C_26_H_33_N_4_O_4_S_2_	529.19377	529.1933	-0.9	-0.47
*b*_FV_-CO	C_25_H_33_N_4_O_3_S_2_	501.19886	501.1994	1.1	0.54
*b*_MF_	C_17_H_24_N_2_S_2_	382.12536	382.1256	0.6	0.24
*b*_FM_	C_12_H_15_N_2_O_2_S	251.08487	251.0843	-2.3	-0.57
*b*_FM_-CO	C_11_H_15_N_2_OS	223.08996	223.0898	-0.7	-0.16

*^a^Measured *m/z* are derived from the MS/MS analysis shown in **Figure 5B**.*

### Specificity of the *Lm* AIP

**FIGURE 6:**
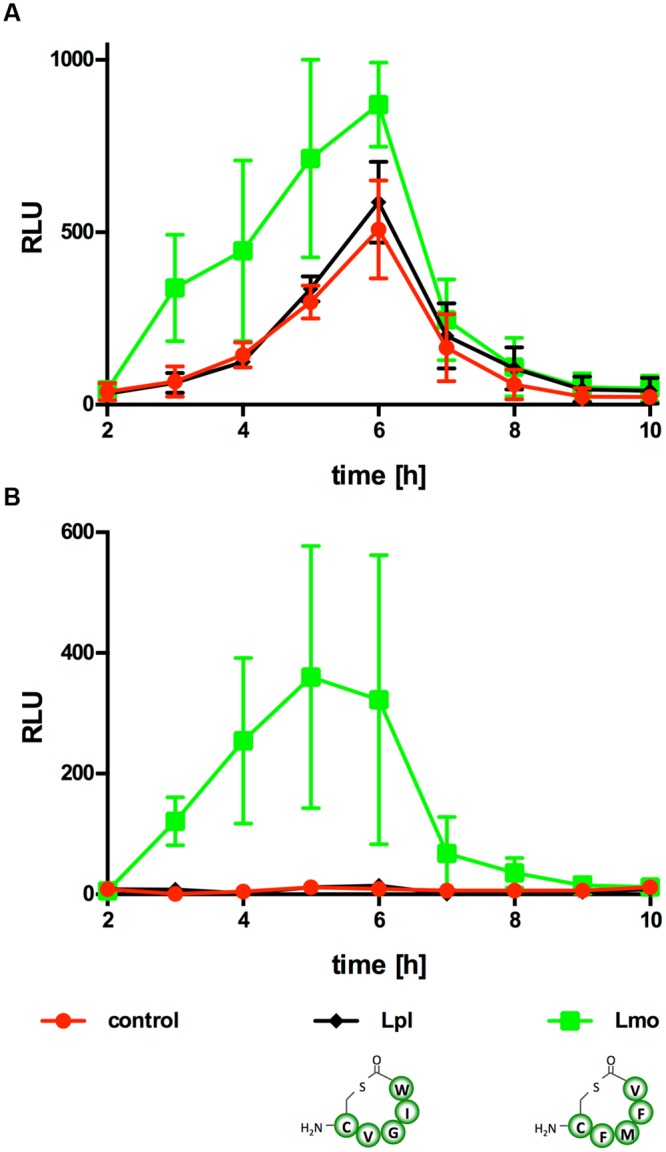
**Luminescence (RLU) of *Lm* EGD-e::pPL2*lux*P_II_**(A)** or Δ*agrD*::pPL2*lux*P_II_**(B)** grown in the presence of the AIP of *Lm* (Lm; i.e., synthetic peptide R5T0) or *L. plantarum* (Lpl).** As controls, bacteria were grown without peptides (control). Values are mean ± standard deviation of three independent experiments. Statistical analysis of RLU values was performed by ANOVA and Bonferroni test to correct for multiple comparisons and results are provided in Supplementary Data Sheet 1.

Known AIPs differ greatly in sequence, length and structure among species and even strains (**Figure 2A**) and different AIPs of *S. aureus* display cross-inhibition (Ji et al., 1997). Similar to the AIP of *Lm*, the AIP of *L. plantarum* is a cyclic pentapeptide yet with a different sequence (Sturme et al., 2005). Further experiments were performed to test if P_II_ activation is specific for the *Lm* AIP or if the *L. plantarum* AIP is also able to activate P_II_ (**Figure 6**). As observed in the previous experiments, R5T0 slightly enhanced P_II_-driven luciferase activity in *Lm* EGD-e::pPL2*lux*P_II_ (**Figure 6A**) and was a potent inducer of P_II_ activity in the AIP-negative reporter strain *Lm* EGD-e Δ*agrD*::pPL2*lux*P_II_. By contrast, in both reporter strains the *L. plantarum* AIP had no effect on P_II_ activity.

## Discussion

Signaling peptides, also referred to as AIPs, are produced by a wide range of Gram-positive microorganisms (Wuster and Babu, 2008) and serve various purposes (Thoendel and Horswill, 2010). The best studied AIP system is the *agr* locus of *S. aureus* and homologous systems have been identified in a variety of Gram-positives (Wuster and Babu, 2008). In *S. aureus*, the *agr* system is a rather global regulatory circuit affecting a large number of genes and different phenotypic traits (Thoendel et al., 2011). Similarly, deletion of *agrD* in *Lm* affects more than 600 genes and phenotypically affects biofilm formation and virulence *in vitro* and *in vivo* (Rieu et al., 2007; Riedel et al., 2009). However, while absence of *agr* signaling is linked with enhanced biofilm formation of *S. aureus* (Vuong et al., 2000), *agr* mutants of *Lm* display reduced biofilm formation under the conditions monitored (Rieu et al., 2007; Riedel et al., 2009).

Previous studies have already indicated that, like the staphylococcal system, the *Lm agr* locus is subject to positive autoregulation involving a diffusible factor, probably the *agrD*-encoded AIP involved in regulation. Transcription levels of the *agr* operon were greatly reduced in *agr*-deficient *Lm* mutants (Rieu et al., 2007; Riedel et al., 2009; Garmyn et al., 2012). Also, the biofilm defect of a Δ*agrD* mutant was complemented when bacteria were grown in the reconstituted culture supernatants of the WT or in the presence of small amounts of WT bacteria (Riedel et al., 2009). The presented results further strengthen the hypothesis that *agrD* encodes a secreted AIP that positively regulates the *agr* system of *Lm*. In the Δ*agrD* mutant, no activity of the *agr* promoter could be observed (**Figure 1A**) and promoter activity was restored when the *agrD*-deficient reporter strain was co-cultured with a strain carrying a plasmid for constitutive expression of *agrBD* (**Figure 1B**).

The presented results provide further evidence that, in *Lm, agrD* actually encodes the propeptide, which is processed released into the extracellular environment where it acts as an AIP. Moreover, our data suggests that the native AIP is a cyclic pentapeptide R5T0 consisting of the amino acids (from N- to C-terminus) Cys, Phe, Met, Phe, Val. A peptide with this structure was found in the culture supernatant of a recombinant *E. coli* strain expressing AgrBD (**Figure 5**) and a synthetic peptide with identical structure was able to potently induce activity of the P_II_ promoter of the *agr* system (**Figure 3**) and to functionally complement the invasion defect in a Δ*agrD* mutant (**Figure 4**).

Induction of luciferase activity in the Δ*agrD* reporter upon co-cultivation with the AIP producing WT strain (**Figure 1B**) indicates that at least some of the AIP must be present in culture supernatants. However, we were unable to identify the native peptide in supernatants of *Lm* EGD-e grown in complex media (brain heart infusion) or modified Welshimer’s broth. This may be explained by the high levels of peptides in brain heart infusion, which makes identification impossible by LC–MS/MS. In modified Welshimer’s broth *Lm* only grows to low final optical densities and thus any secreted peptide will also be present at low concentrations especially when subject to positive autoregulation and fully induced only at high cell densities. Further studies will be needed to quantify actual AIP concentrations in culture supernatants and the threshold required to activate PII and target gene regulation.

Interestingly, four different synthetic peptides with a five-membered thiolactone ring and varying tail length had inhibitory activity on the *agr* promoter in the WT reporter strain, which itself is able to produce the native AIP. Since *agr* mutants of *Lm* display attenuated virulence (Autret et al., 2003; Riedel et al., 2009), this suggests that these peptides are antagonists of the native AIP and may represent a potential supplementary or alternative therapeutic approach as proposed for *S. aureus* and other pathogens (Gray et al., 2013). Interestingly, they also exhibited P_II_ activation in the Δ*agrD* reporter to varying degrees. This may indicate that these peptides compete with R5T0 or the native AIP for binding to the receptor but their affinity and/or activity is lower. Thus, of the four candidate peptides, the best antagonist of the native AIP is probably R5T2, which efficiently blocks P_II_ activity in the WT but activates luminescence inly marginally in the mutant reporter.

A striking difference between the *agr* systems of *S. aureu*s and *Lm* is the structural diversity of the AIPs. Within the species *S. aureus*, four specificity groups of strains with different AIP are found and these groups show cross-inhibition (Novick and Geisinger, 2008). By contrast, the AgrD propeptides of the genus *Listeria* are rather conserved and the species *Lm, L. innocua, L. ivanovii, L. welshimeri, L. seeligeri*, and *L. marthii* have identical (predicted) AIP sequences (Supplementary Figure S3A) suggesting cross-reactivity. Moreover, phylogenetic analysis based on 16S rRNA gene sequences reveals that *Listeria sp.* that share identical AIP sequences form a cluster that separates from the other species indicating that they are more closely related (Supplementary Figure S3B).

With the exception of *C. acetobutylicum*, phylogenetic trees calculated using concatenate AgrA, AgrB, AgrC, and AgrD sequences are in line with trees inferred from 16S sequences (Wuster and Babu, 2008). This suggests that *agr* systems are generally inherited vertically. It has been proposed that *C. acetobutylicum*, whose AgrD sequence is almost identical to that of *Listeriaceae*, is the only known case of horizontal transfer of an *agr* system (Wuster and Babu, 2008). Further experimental data comparing the *Lm* AIP with the AIP of *L. plantarum*, which also consist of a five cyclic pentapeptide although with different aa composition, indicates that the *Lm agr* system is specific for the AIP of those *Listeria* sp. that share a conserved AgrD sequence but does not respond to the cyclic pentapeptide AIPs of other organisms. This also suggests that intervention strategies based on antagonistic peptides targeting the *agr* systems of *Lm* (and other organisms) are specific for organisms with identical AIPs.

In summary, the presented data shows that the *agrD* of *Lm* EGD-e encodes a secreted peptide consisting of a five-membered thiolactone ring, which has autoinducing activity. Moreover, the identification of several synthetic peptides with antagonistic activity proposes a potential option to treat *Lm* infections or inhibit biofilm formation as suggested by others previously.

## Author Contributions

CR conceived the study. MZ, MW, and AS-K carried out experiments. MZ, AS-K, BB, and CR analyzed data. MZ, AS-K, BB, and CR drafted the manuscript and all the authors contributed to preparing the final version of the manuscript. All authors read and approved the final manuscript.

## Conflict of Interest Statement

The authors declare that the research was conducted in the absence of any commercial or financial relationships that could be construed as a potential conflict of interest.

## References

[B1] Autret N. Raynaud C. Dubail I. Berche P. Charbit A. (2003). Identification of the agr locus of *Listeria monocytogenes*: role in bacterial virulence. *Infect. Immun.* 71 4463–4471. 10.1128/IAI.71.8.4463-4471.2003 12874326 PMC166014

[B2] Bécavin C. Bouchier C. Lechat P. Archambaud C. Creno S. Gouin E. (2014). Comparison of widely used *Listeria monocytogenes* strains EGD, 10403S, and EGD-e highlights genomic variations underlying differences in pathogenicity. *MBio* 5 e969–14. 10.1128/mBio.00969-14 PMC3977354 24667708

[B3] Bron P. A. Monk I. R. Corr S. C. Hill C. Gahan C. G. M. (2006). Novel luciferase reporter system for in vitro and organ-specific monitoring of differential gene expression in *Listeria monocytogenes*. *Appl. Environ. Microbiol.* 72 2876–2884. 10.1128/AEM.72.4.2876-2884.2006 16597994 PMC1449049

[B4] Cook L. C. Federle M. J. (2014). Peptide pheromone signaling in *Streptococcus* and *Enterococcus*. *FEMS Microbiol. Rev.* 38 473–492. 10.1111/1574-6976.12046 24118108 PMC4103628

[B5] Ferreira V. Wiedmann M. Teixeira P. Stasiewicz M. J. (2014). *Listeria monocytogenes* persistence in food-associated environments: epidemiology, strain characteristics, and implications for public health. *J. Food Prot.* 77 150–170. 10.4315/0362-028X.JFP-13-150 24406014

[B6] Freitag N. E. Port G. C. Miner M. D. (2009). *Listeria monocytogenes* – from saprophyte to intracellular pathogen. *Nat. Rev. Microbiol.* 7 623–628. 10.1038/nrmicro2171 19648949 PMC2813567

[B7] Fujii T. Ingham C. Nakayama J. Beerthuyzen M. Kunuki R. Molenaar D. (2008). Two homologous Agr-like quorum-sensing systems cooperatively control adherence, cell morphology, and cell viability properties in *Lactobacillus plantarum* WCFS1. *J. Bacteriol.* 190 7655–7665. 10.1128/JB.01489-07 18805979 PMC2583610

[B8] Gahan C. G. M. Hill C. (2014). *Listeria monocytogenes*: survival and adaptation in the gastrointestinal tract. *Front. Cell. Infect. Microbiol.* 4:9 10.3389/fcimb.2014.00009 PMC3913888 24551601

[B9] Garmyn D. Augagneur Y. Gal L. Vivant A.-L. Piveteau P. (2012). *Listeria monocytogenes* differential transcriptome analysis reveals temperature-dependent Agr regulation and suggests overlaps with other regulons. *PLoS ONE* 7:e43154 10.1371/journal.pone.0043154 PMC3443086 23024744

[B10] Gray B. Hall P. Gresham H. (2013). Targeting agr- and agr-Like quorum sensing systems for development of common therapeutics to treat multiple gram-positive bacterial infections. *Sensors (Basel)* 13 5130–5166. 10.3390/s130405130 23598501 PMC3673130

[B11] Guariglia-Oropeza V. Orsi R. H. Yu H. Boor K. J. Wiedmann M. Guldimann C. (2014). Regulatory network features in *Listeria monocytogenes*-changing the way we talk. *Front. Cell. Infect. Microbiol.* 4:14 10.3389/fcimb.2014.00014 PMC3924034 24592357

[B12] Ji G. Beavis R. Novick R. P. (1997). Bacterial interference caused by autoinducing peptide variants. *Science* 276 2027–2030. 10.1126/science.276.5321.2027 9197262

[B13] Kalkum M. Lyon G. J. Chait B. T. (2003). Detection of secreted peptides by using hypothesis-driven multistage mass spectrometry. *Proc. Natl. Acad. Sci. U.S.A.* 100 2795–2800. 10.1073/pnas.0436605100 12591958 PMC151420

[B14] Lauer P. Chow M. Y. N. Loessner M. J. Portnoy D. A. Calendar R. (2002). Construction, characterization, and use of two *Listeria monocytogenes* site-specific phage integration vectors. *J. Bacteriol.* 184 4177–4186. 10.1128/JB.184.15.4177-4186.2002 12107135 PMC135211

[B15] Mandin P. Repoila F. Vergassola M. Geissmann T. Cossart P. (2007). Identification of new noncoding RNAs in *Listeria monocytogenes* and prediction of mRNA targets. *Nucleic Acids Res.* 35 962–974. 10.1093/nar/gkl1096 17259222 PMC1807966

[B16] McGrath S. Fitzgerald G. F. van Sinderen D. (2001). Improvement and optimization of two engineered phage resistance mechanisms in *Lactococcus lactis*. *Appl. Environ. Microbiol.* 67 608–616. 10.1128/AEM.67.2.608-616.2001 11157223 PMC92627

[B17] Mellin J. R. Cossart P. (2012). The non-coding RNA world of the bacterial pathogen *Listeria monocytogenes*. *RNA Biol.* 9 372–378. 10.4161/rna.19235 22336762

[B18] Monk I. R. Gahan C. G. M. Hill C. (2008). Tools for functional postgenomic analysis of *Listeria monocytogenes*. *Appl. Environ. Microbiol.* 74 3921–3934. 10.1128/AEM.00314-08 18441118 PMC2446514

[B19] Nakayama J. Cao Y. Horii T. Sakuda S. Akkermans A. D. de Vos W. M. (2001). Gelatinase biosynthesis-activating pheromone: a peptide lactone that mediates a quorum sensing in *Enterococcus faecalis*. *Mol. Microbiol.* 41 145–154. 10.1046/j.1365-2958.2001.02486.x 11454207

[B20] Novick R. P. Geisinger E. (2008). Quorum sensing in staphylococci. *Annu. Rev. Genet.* 42 541–564. 10.1146/annurev.genet.42.110807.091640 18713030

[B21] Olson M. E. Todd D. A. Schaeffer C. R. Paharik A. E. Van Dyke M. J. Büttner H. (2014). *Staphylococcus epidermidis* agr quorum-sensing system: signal identification, cross talk, and importance in colonization. *J. Bacteriol.* 196 3482–3493. 10.1128/JB.01882-14 25070736 PMC4187671

[B22] Otto M. Süssmuth R. Jung G. Götz F. (1998). Structure of the pheromone peptide of the *Staphylococcus epidermidis* agr system. *FEBS Lett.* 424 89–94. 10.1016/S0014-5793(98)00145-8 9537521

[B23] Paliy O. Gunasekera T. S. (2007). Growth of E. coli BL21 in minimal media with different gluconeogenic carbon sources and salt contents. *Appl. Microbiol. Biotechnol.* 73 1169–1172. 10.1007/s00253-006-0554-8 16944129

[B24] Qin X. Singh K. V. Weinstock G. M. Murray B. E. (2001). Characterization of fsr, a regulator controlling expression of gelatinase and serine protease in *Enterococcus faecalis* OG1RF. *J. Bacteriol.* 183 3372–3382. 10.1128/JB.183.11.3372-3382.2001 11344145 PMC99635

[B25] Qiu X.-Q. Wang H. Lu X.-F. Zhang J. Li S.-F. Cheng G. (2003). An engineered multidomain bactericidal peptide as a model for targeted antibiotics against specific bacteria. *Nat. Biotechnol.* 21 1480–1485. 10.1038/nbt913 14625561

[B26] Queck S. Y. Jameson-Lee M. Villaruz A. E. Bach T.-H. L. Khan B. A. Sturdevant D. E. (2008). RNAIII-independent target gene control by the agr quorum-sensing system: insight into the evolution of virulence regulation in *Staphylococcus aureus*. *Mol. Cell* 32 150–158. 10.1016/j.molcel.2008.08.005 18851841 PMC2575650

[B27] Riedel C. U. Monk I. R. Casey P. G. Waidmann M. S. Gahan C. G. M. Hill C. (2009). AgrD-dependent quorum sensing affects biofilm formation, invasion, virulence and global gene expression profiles in *Listeria monocytogenes*. *Mol. Microbiol.* 71 1177–1189. 10.1111/j.1365-2958.2008.06589.x 19154329

[B28] Rieu A. Weidmann S. Garmyn D. Piveteau P. Guzzo J. (2007). Agr system of *Listeria monocytogenes* EGD-e: role in adherence and differential expression pattern. *Appl. Environ. Microbiol.* 73 6125–6133. 10.1128/AEM.00608-07 17675424 PMC2075002

[B29] Steiner E. Scott J. Minton N. P. Winzer K. (2012). An agr quorum sensing system that regulates granulose formation and sporulation in *Clostridium acetobutylicum*. *Appl. Environ. Microbiol.* 78 1113–1122. 10.1128/AEM.06376-11 22179241 PMC3273008

[B30] Sturme M. H. J. Nakayama J. Molenaar D. Murakami Y. Kunugi R. Fujii T. (2005). An agr-like two-component regulatory system in *Lactobacillus plantarum* is involved in production of a novel cyclic peptide and regulation of adherence. *J. Bacteriol.* 187 5224–5235. 10.1128/JB.187.15.5224-5235.2005 16030216 PMC1196011

[B31] Thoendel M. Horswill A. R. (2010). Biosynthesis of peptide signals in gram-positive bacteria. *Adv. Appl. Microbiol.* 71 91–112. 10.1016/S0065-2164(10)71004-2 20378052 PMC6916668

[B32] Thoendel M. Kavanaugh J. S. Flack C. E. Horswill A. R. (2011). Peptide signaling in the staphylococci. *Chem. Rev.* 111 117–151. 10.1021/cr100370n 21174435 PMC3086461

[B33] Toledo-Arana A. Dussurget O. Nikitas G. Sesto N. Guet-Revillet H. Balestrino D. (2009). The *Listeria* transcriptional landscape from saprophytism to virulence. *Nature* 459 950–956. 10.1038/nature08080 19448609

[B34] Vivant A.-L. Garmyn D. Gal L. Hartmann A. Piveteau P. (2015). Survival of *Listeria monocytogenes* in soil requires agra-mediated regulation. *Appl. Environ. Microbiol.* 81 5073–5084. 10.1128/AEM.04134-14 26002901 PMC4495223

[B35] Vivant A.-L. Garmyn D. Piveteau P. (2013). *Listeria monocytogenes*, a down-to-earth pathogen. *Front. Cell. Infect. Microbiol.* 3:87 10.3389/fcimb.2013.00087 PMC3842520 24350062

[B36] Vuong C. Saenz H. L. Götz F. Otto M. (2000). Impact of the agr quorum-sensing system on adherence to polystyrene in *Staphylococcus aureus*. *J. Infect. Dis.* 182 1688–1693. 10.1086/317606 11069241

[B37] Williams T. Bauer S. Beier D. Kuhn M. (2005). Construction and characterization of *Listeria monocytogenes* mutants with in-frame deletions in the response regulator genes identified in the genome sequence. *Infect. Immun.* 73 3152–3159. 10.1128/IAI.73.5.3152-3159.2005 15845524 PMC1087338

[B38] Wurtzel O. Sesto N. Mellin J. R. Karunker I. Edelheit S. Bécavin C. (2012). Comparative transcriptomics of pathogenic and non-pathogenic *Listeria* species. *Mol. Syst. Biol.* 8 583 10.1038/msb.2012.11 PMC3377988 22617957

[B39] Wuster A. Babu M. M. (2008). Conservation and evolutionary dynamics of the agr cell-to-cell communication system across firmicutes. *J. Bacteriol.* 190 743–746. 10.1128/JB.01135-07 17933897 PMC2223712

