# Supplementary material for: Identification of the agr Peptide of Listeria monocytogenes
Source: Front Microbiol. 2016 Jun 22;7:989. doi: 10.3389/fmicb.2016.00989 (PMC4916163; doi:10.3389/fmicb.2016.00989)
Supplement: Supplementary file 1 [file Presentation_1.PDF]

Supplementary Material

Identification of the *agr* peptide of *Listeria monocytogenes*

Marion Zetzmann[1], Andrés Sánchez-Kopper[2,3], Mark S. Waidmann[1], Bastian Blombach[2], and Christian U. Riedel[1,*]

*** Correspondence:** Dr. Christian Riedel, Institute of Microbiology and Biotechnology, University of Ulm, Albert-Einstein-Allee 11, 89081 Ulm, Germany, christian.riedel@uni-ulm.de

1 Supplementary Figures

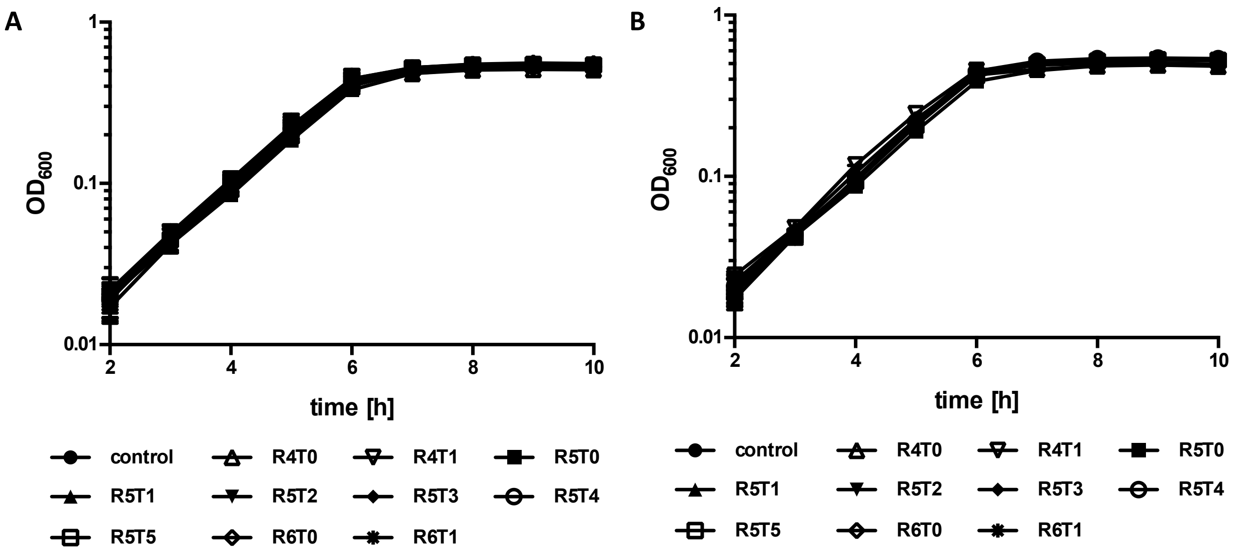

Supplementary Figure 1: Growth of *Lm* EGD-e::pPL2*lux*P_{II} (A) or Δ*agrD*::pPL2*lux*P_{II} (B) in BHI at 30 °C in the presence of different synthetic peptides at a final concentration of 5 µM. Values are mean ± standard deviation of three independent cultures per strain.

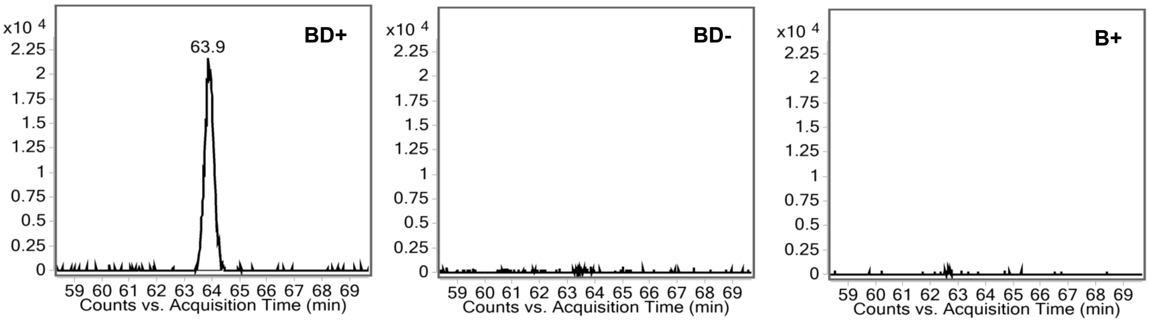

Supplementary Figure 2: Extracted ion chromatograms performed on culture supernatant of *E. coli* BL21 DE3 pET29a_*agrBD* after induction with IPTG (BD+; left panel). Supernatants of *E. coli* BL21 DE3 pET29a_*agrBD* before induction (BD-; middle panel) and *E. coli* BL21 DE3 pET29a_*agrB* (B+; right panel) were analyzed as controls.

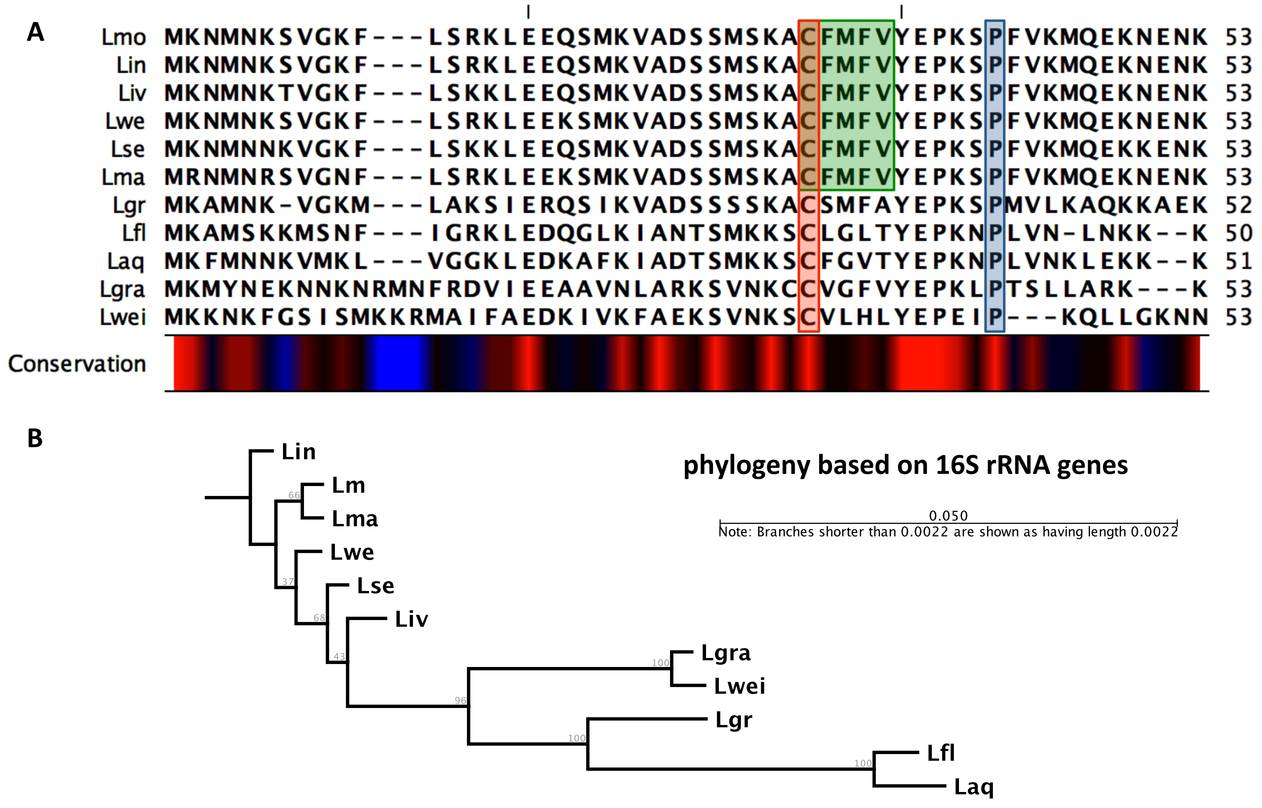

Supplementary Figure 3: Alignment of AgrD amino acid sequences available the NCBI protein databases (A) and phylogeny of *Listeria sp.* using 16S rRNA gene sequences (B). In silico analysis was performed using CLC Workbench (version 7.0). Phylogenetic analysis was performed on 16S rRNA gene sequences obtained from the NCBI nucleotide database and truncated to encompass the central 1390 nucleotides for which sequence information was available for all species analyzed. The phylogenetic tree was constructed by calculating the Maximum Likelihood Phylogeny using the Neighbour Joining method and Jukes Cantor nucleotide substitution. Bootstrap analysis was performed by running 100 iterations. Abbreviations of the *Listeria sp.* and Accession numbers of the corresponding AgrD sequences: *L. monocytogenes* (Lmo; CAC98264), *L. innocua* (Lin; CAC95275), *L. invanovii* (Liv: AHI546872), *L. welshimeri* (Lwe; CAK19458), *L. seeligeri* (Lse; EFS04697), *L. marthii* (Lma; EFR89221), *L. grayi* (Lgr; EUJ28600), *L. fleischmannii* (Lfl; EMG28258), *L. aquatica* (Laq; EUJ17415), *L. grandensis* (Lgra; EUJ22546), *L. weihenstephanensis* (Lwei; EUJ36606). Highlighted are the conserved central cystein residue (red-shaded box), identical putative AIP sequences (green-shaded box) and proline residue required for processing and cleavage (blue-shaded box).
